# Nontarget effects of foliar fungicide application on the rhizosphere: diversity of *nifH* gene and nodulation in chickpea field

**DOI:** 10.1111/j.1365-2672.2012.05262.x

**Published:** 2012-05

**Authors:** C Yang, C Hamel, V Vujanovic, Y Gan

**Affiliations:** 1 Semiarid Prairie Agricultural Research Centre, AAFCSwift Current, SK, Canada; 2 Food and Bioproducts Sciences, University of SaskatchewanSaskatoon, SK, Canada

**Keywords:** bacteria, biological nitrogen fixation, fungicide, nontarget effects, rhizosphere

## Abstract

**Aims:**

This study explores nontarget effects of fungicide application on field-grown chickpea.

**Methods and Results:**

Molecular methods were used to test the effects of foliar application of fungicide on the diversity and distribution of *nifH* genes associated with two chickpea cultivars and their nodulation. Treatments were replicated four times in a split-plot design in the field, in 2008 and 2009. Chemical disease control did not change the richness of the *nifH* genes associated with chickpea, but selected different dominant *nifH* gene sequences in 2008, as revealed by correspondence analysis. Disease control strategies had no significant effect on disease severity or *nifH* gene distribution in 2009. Dry weather conditions rather than disease restricted plant growth that year, suggesting that reduced infection rather than the fungicide is the factor modifying the distribution of *nifH* gene in chickpea rhizosphere. Reduced nodule size and enhanced N_2_-fixation in protected plants indicate that disease control affects plant physiology, which may in turn influence rhizosphere bacteria. The genotypes of chickpea also affected the diversity of the *nifH* gene in the rhizosphere, illustrating the importance of plant selective effects on bacterial communities.

**Conclusions:**

We conclude that the chemical disease control affects nodulation and the diversity of *nifH* gene in chickpea rhizosphere, by modifying host plant physiology. A direct effect of fungicide on the bacteria cannot be ruled out, however, as residual amounts of fungicide were found to accumulate in the rhizosphere soil of protected plants.

**Significance and Impact of the Study:**

Systemic nontarget effect of phytoprotection on *nifH* gene diversity in chickpea rhizosphere is reported for the first time. This result suggests the possibility of manipulating associative biological nitrogen fixation in the field.

## Introduction

Nitrogen limits plant growth in many ecosystems (Fiore *et al.* 2010). Biological nitrogen fixation (BNF) makes an important contribution to soil nitrogen (Zielke *et al.* 2005; Zhao *et al.* 2010) and improves plant productivity. Much research was devoted to understand the mechanisms of BNF in diazotrophs (Kessler and Leigh 1999; Petrova *et al.* 2000; Bashan and de-Bashan 2010; Oliveira *et al.* 2010) because of the importance of their contribution to the biosphere. Diazotrophs possess the enzymes nitrogenase and nitrogenase reductase carrying out N_2_-fixation, that is, the reduction in N_2_ into NH_3_. These N_2_-fixing bacteria are diverse taxonomically and metabolically, but can be classified into three functional groups (Bürgmann *et al.* 2004). The free-living N_2_-fixing bacteria contribute a relatively small proportion of the N input in ecosystems, because of the high energy requirement of the process. The associative N_2_-fixing bacteria typically live on plant roots surface and can be quite active when fuelled by rhizodepositions. Symbiotic N_2_-fixing bacteria trigger the formation of specialized organs such as root nodules within plant tissues and can fix considerable amounts of N_2_. Symbiotic N_2_-fixing bacteria are largely associated with leguminous plants (Lindström *et al.* 2010) and N_2_-fixing leguminous crops are widely used to input BNF in agro-ecosystems throughout the world.

Denitrification of nitrogenous fertilizer residues into N_2_O was identified as the main source of greenhouse gas emissions from farming activities (Janzen *et al.* 2006; Dyer *et al.* 2010; van Groenigen *et al.* 2010). Improved cropping systems involving N_2_-fixing crops in rotations can reduce the amount of greenhouse gas emissions and the environmental impact of agriculture (Gan *et al.* 2011). Therefore, N_2_-fixing bacteria and BNF in cultivated fields are triggering much research interest.

Chickpea (*Cicer arietinum* L.) is the third most important leguminous crops worldwide. It is grown in the Mediterranean countries, Middle East, West Asia, Mexico and elsewhere (Kyei-Boahen *et al.* 2002; Pande *et al.* 2005; Millan *et al.* 2006). Chickpea is widely grown in rotation with wheat in southwest Saskatchewan and southeast Alberta, the driest part of the Canadian Prairie, where low precipitation, high diurnal temperature fluctuation and sufficient heat lead to high-quality grain. Chickpea could be an important source of nitrogen in wheat-based cropping systems of semi-arid regions of the world, but nodulation in this crop is sometimes reduced (Broughton and Perret 1999). Relatively few studies have examined the diversity of N_2_-fixing bacteria in field-grown chickpea (Laranjo *et al.* 2008). *Mesorhizobium ciceri* and *Mesorhizobium mediterraneum* are known to nodulate chickpea (Nour *et al.* 1994, 1995). A later report showed a few more species able to nodulate chickpea (Laranjo *et al.* 2004); however, these results remain controversial (Laranjo *et al.* 2004; Rivas *et al.* 2007).

The poor reliability of nodulation in chickpea may be related to cropping practices rather than to plant genetics. Fungicides are used abundantly in chickpea crops to control Ascochyta blight, a devastating disease of this crop (Gan *et al.* 2006). Pesticide use may adversely affect agriculturally important micro-organisms, including N_2_-fixing bacteria, and reduce the performance of agroecosystems (Gaind *et al.* 2007). A close look at the effect of fungicide application on N_2_-fixing bacteria in chickpea fields could help explain the variation in BNF activity observed in this crop and lead to the design of more sustainable cropping systems.

Molecular techniques have been used in research on N_2_-fixing bacteria to resolve many important problems associated with traditional cultural methods (Hugenholtz *et al.* 1998). Among molecular tools, PCR-based profiling methods such as restriction fragment length polymorphism (RFLP) (Bürgmann *et al.* 2004) and denaturing gradient gel electrophoresis (DGGE) (Bürgmann *et al.* 2005) have been used to analyse the diversity of N_2_-fixing bacterial communities. Nitrogenase reductase structural gene *nifH* (Howard and Rees 1996) was successfully used as a marker gene for BNF (Bürgmann *et al.* 2004). We adopted a PCR-DGGE protocol using *nifH* as a target to: (1) improve knowledge on the N_2_-fixing bacterial diversity in field-grown chickpea rhizosphere and (2) define the effect of foliar disease control on chickpea rhizobacterial community.

## Materials and methods

### Experimental design and treatment application

A two-factor field experiment with split-plot design and four replicates was conducted in 2008 and 2009 at different locations of the Semiarid Prairie Agricultural Research Centre, near Swift Current, SK, Canada (latitude 50°18′N; longitude 107°41′W). The soil contained 3·6 kg ha^−1^ mineral N, 21·8 kg ha^−1^ sodium bicarbonate extractable P and 283 kg ha^−1^ available K in 2008, and 3·1 kg ha^−1^ mineral N, 12·6 kg ha^−1^ sodium bicarbonate extractable P and 210 kg ha^−1^ available K in 2009. The climatic conditions were drier in 2009 than 2008. Average precipitation during the growing season, that is, from April to September, was 59·3 mm month^−1^ in 2008 and 35·6 mm month^−1^ in 2009. Treatments consisted in a nontreated control and four different fungal disease control strategies (Table 1), involving Bravo® (Syngenta Crop Protection Canada Inc., Guelph, ON, Canada, a.i. chlorothalonil) and Headline® Duo (BASF Canada Inc., Mississauga, ON, Canada, a.i. pyraclostrobin and boscalid), two fungicides commonly used to control Ascochyta blight in chickpea fields. These treatments were applied to two chickpea cultivars, CDC Luna and CDC Vanguard, representing two main types of chickpea, Kabuli and Desi, which differ in seed size, shape, colour and nutrients content (Iqbal *et al.* 2006; Maheri-Sis *et al.* 2008). Nitragin Soil Implant + GC Peat-based Granular Inoculant, which contains a minimum of 100 million (1 × 10^8^) viable cells of *M. ciceri* per gram of product, was applied at 5·6 kg ha^−1^. The *nifH* gene sequences in this commercial inoculant were verified through DNA extraction, cloning and sequencing, using the procedure described below. The inoculant contained two *nifH* gene sequences. One was 97% similar to a *M ciceri* (GenBank no. EU267715.1) and another was 97% similar to *Bradyrhizobium* sp. (GenBank no. CP000494.1).

**Table 1:** Timing of application and type of fungicide making up the foliar disease control treatments used in the experiment

	Chickpea growth stage				
	
Treatment	Seedling	Vegetative	Early-flower	Mid-flower	Podding
Control (C)	/*	/	/	/	/
I	Headline® Duo†	/	Headline® Duo	/	/
II	Headline® Duo	Bravo®	Headline® Duo	/	/
III	Headline® Duo	Bravo®	Headline® Duo	Bravo®	Bravo®

* Nothing was applied.

† Recommended rates of 1·0 kg a.i. ha^−1^ chlorothalonil (Bravo) and 100 g a.i. ha^−1^ pyraclostrobin and 240 g a.i. ha^−1^ boscalid (Headline® Duo) were used at each application.

### Soil sampling

Rhizosphere soil samples were taken at chickpea harvest time in September of 2008 and 2009. Two soil cores (0–7·5 cm depth) were taken directly on the crop row using a 5-cm diameter manual soil sampler after sweeping away plant debris and pooled to yield one composite sample per plot. Samples were brought to the laboratory, sieved through 2 mm and placed in sealed plastic bags at −20°C until molecular analysis.

### Nodule sampling

Nodules were sampled when BNF usually peaks, that is, 1 week after chickpea early-flowering stage. As plots assigned to treatment II and treatment III were still treated exactly the same at that time, nodulation was not assessed in plots receiving treatment III. Five plants from each plot were removed using a shovel to minimize root disturbance and brought to the laboratory. Their roots were carefully cleaned with tap water to remove adhering soil and dried with paper towels before randomly collecting five nodules from each plant (Rice and Clayton 1996). A nodulation score test based on internal colour and size of nodules was then applied (Rice and Clayton 1996), using the pools of 25 nodules randomly collected from each plot.

### Measurement of fixed nitrogen

The ^15^N dilution technique was used to measure the amount of nitrogen fixed by chickpea under different treatments, using barley (*Hordeum vulgare* L.) as the nonfixing control plant (Jensen 1986). For this, a barley plot was planted beside each chickpea plot. ^15^NH_4_^15^NO_3_ (10 atom%, Icon Isotopes, http://www.iconisotopes.com) was applied to both chickpea and barley microplots after plant emergence. Whole plants were collected at harvest time, taken back to the laboratory, cleaned with tap water to remove the soil attached on their surface, dried at 45°C until constant weights and finely ground. Plant nitrogen concentration and ^15^N-to-^14^N ratio were measured by mass spectrometry (V.G. Isotech, Middlewich, UK). The percentage and amount of nitrogen derived from air were calculated as: 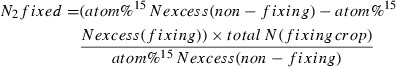


(Fried and Middelboe 1977).

### Molecular analysis of *nifH* gene diversity in chickpea rhizosphere

Raw DNA was extracted from chickpea rhizosphere soil using UltraClean Soil DNA Isolation Kit (MO BIO Laboratories, Inc., Carlsbad, CA, USA), according to the manufacturers’ instruction, and diluted 20 times before PCR amplification of a fragment (*c.* 450 bp) of the gene *nifH* using primers PloR/PloF (Poly *et al.* 2001). The PCR products were used as templates in a subsequent PCR using the same protocol except for the primers, which were PloR and PloF-GC, that is, PloF with a GC clamp at the 5′ end. This amplification produced fragments of *c.* 500 bp, which were used to construct a clone library and DGGE markers. UltraPure™ DNase/RNase-Free Distilled Water (Cat no. 10977015; Invitrogen, Burlington, ON, Canada) was included in PCR instead of DNA template as negative control to exclude any risk of false DNA amplification.

A clone library of all the *nifH* gene sequences obtained from soil samples was created by pooling the PCR products amplified with primers PloR/PloF from soil samples (Renker *et al.* 2006). The DNA fragments were cloned into *Escherichia coli* (strain TOP 10) using the TOPO TA Cloning Kit (Cat no. K4575-J10, Invitrogen) following the manufacturer’s instructions. The transformed cells were plated onto solid Luria–Bertani (LB) medium containing ampicillin (50 μg ml^−1^), incubated overnight at 37°C, then transferred into a 96-well plate filled with liquid LB medium and sent for sequencing at the Plant Biotechnology Institute of the National Research Council of Canada, in Saskatoon, SK. The N_2_-fixing bacteria associated with our experimental chickpea plants were identified based on the similarity of their *nifH* gene sequence to sequences deposited in GenBank, using the online program blast. Positive clones were subjected to PCR amplification using primer pair PloR/PloF-GC as mentioned above, and 10 μl of PCR product of each clone was submitted to DGGE, as described below, to locate a distinct migration position for each clone on the gel. Then, 10 μl of PCR product of each clone was pooled. This DGGE marker mix was loaded (40 μl) into a lane on each gel for the identification of the bands produced from experimental samples. All DNAs were stored at −20°C prior to analysis.

A DGGE protocol (Ma *et al.* 2005) was used to separate 20 μl of PCR products from each plot. Gels contained 6% (w/v) polyacrylamide (37: 1 acrylamide/bis-acrylamide). The linear gradient used varied from 35 to 65% denaturant, where 100% denaturing acrylamide was defined as containing 7 mol l^−1^ urea and 40% (v/v) formamide. A 4-ml stacking gel containing no denaturants was added before polymerization was complete (*c.* 2 h). All DGGE separations were performed in a Dcode Universal Mutation Detection System (Bio-Rad Laboratories, Hercules, CA, USA) at a constant temperature of 60°C. After 10 min at 75 V, the voltage was lowered to 60 V for an additional 16 h. Gels were stained in 1× Tris/acetic acid/EDTA buffer (TAE) containing 4 μl SYBR Safe DNA gel stain (Invitrogen) per 10 ml and visualized by UV illumination. Gel images were digitally captured by an Olympus digital camera (SP-500UZ) in Multimage Light Cabinet (Alpha Innotech Corp., San Leandro, CA, USA) using a Sybr Safe filter.

### Statistical analysis

Linear regression analysis was used to verify the relationship between chickpea yield, fixed nitrogen and disease severity using systat 12. The *nifH* sequences detected in this study were aligned by BioEdit sequence alignment editor software (ver. 7.0.9.0.) using Clustal W multiple alignment algorithm. The diversity of *nifH* gene associated with the chickpea crops submitted to the different experimental treatments, as revealed by sequence profiling, was analysed by MultiResponse Permutation Procedure (MRPP) using PC-ORD and correspondence analysis (CA) using systat 12. Difference of nodulation scores was detected by anova using systat 12. The Shapiro–Wilk test was used to verify the normality of distribution and homogeneity of variance prior to anova. The Wilks’ Lambda test was used to detect significant treatment effects at 5% level in anova using systat 12.

## Results

### Diversity of *nifH* gene fragments

**Figure 1:**
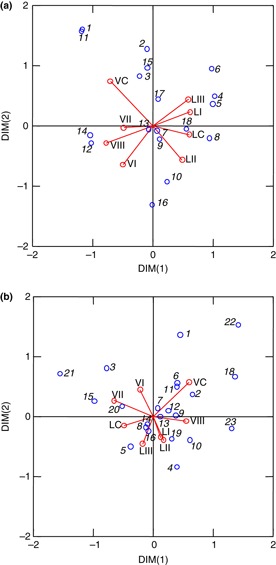
Correspondence analysis (CA) of relationships between disease control treatments and identified dominant N_2_-fixing bacteria in the rhizosphere of both chickpea cultivars in 2008 (a) and 2009 (b), as revealed by *nifH* gene. C: control; I, II and III: increasing intensity of fungicide application; V: CDC Vanguard; L: CDC Luna; Numbers correspond to the identified N_2_-fixing bacteria shown in Table 2. *P*=0·014 in 2008, *P*>0·05 in 2009, *N* = 32.

A total of 23 different *nifH* gene sequences were detected by the PCR-DGGE analysis method (Table 2), and *nifH* sequences closely affiliated to *M. ciceri* were found. However, eight sequences were related to other symbiotic and nonsymbiotic genera, and eleven sequences showed close similarity to uncultured species, revealing a high diversity of *nifH* gene in chickpea rhizosphere soil. In 2008, significant effects of cultivar (*P*<0·001) on community structure (Fig. 1a) were detected by MRPP analysis, revealing a selective effect of chickpea genotype on the diversity of rhizosphere *nifH* gene. No significant effects of genotypes on *nifH* gene diversity were found in 2009 (Fig. 1a).

**Table 2:** Identity of the N_2_-fixing bacteria living in chickpea rhizosphere, according to blast results

Sequence designation	Year	GenBank accession no. for closest match	Closest match in GenBank by blast*
1	2008 and 2009	AY583643.1	Uncultured bacterium clone SJ14 dinitrogenase reductase (*nifH*) gene, partial cds (98%)
2	2008 and 2009	AY819584.1	Uncultured bacterium clone M1b-77 dinitrogenase reductase (*nifH*) gene, partial cds (97%)
3	2008 and 2009	AB188121.1	*Azohydromonas australica nifH* gene for iron protein of nitrogenase, partial cds, strain:IAM 12664 (97%)
4	2008 and 2009	CP000494.1†	*Bradyrhizobium* sp. BTAi1, complete genome (97%)
5	2008 and 2009	AY196375.1	Uncultured nitrogen-fixing bacterium clone b1-HA3-7 nitrogenase iron protein (*nifH*) gene, partial cds (100%)
6	2008 and 2009	DQ995922.1	Uncultured nitrogen-fixing bacterium clone 57 dinitrogenase reductase (*nifH*) gene, partial cds (98%)
7	2008 and 2009	AB217474.1	*Sphingomonas azotifigens nifH* gene for dinitrogenase reductase subunit, partial cds (99%)
8	2008 and 2009	AY360976.1	Uncultured bacterium cluster *O NifH* (*nifH*) gene, partial cds (97%)
9	2008 and 2009	GU201868.1	*Rhizobium leguminosarum* strain Qtx-10-1 *NifH*-like (*nifH*) gene, partial sequence (97%)
10	2008 and 2009	AB542349.1	*Azospirillum* sp. TSA20c *nifH* gene for nitrogenase reductase, partial cds, strain: TSA20c (97%)
11	2008 and 2009	AM110711.1	*Azorhizobium caulinodans* partial *nifH* gene for putative nitrogenase, isolate T1 2 (98%)
12	2008 and 2009	EU267715.1†	*Mesorhizobium ciceri* strain USDA 3378 nitrogenase iron protein (*nifH*) gene, partial cds (97%)
13	2008 and 2009	DQ995918.1	Uncultured nitrogen-fixing bacterium clone 50 dinitrogenase reductase (*nifH*) gene, partial cds (98%)
14	2008 and 2009	GQ167280.1	*Mesorhizobium mediterraneum* strain USDA 3392 *NifH* (*nifH*) gene, partial cds; (99%)
15	2008 and 2009	AY583648.1	Uncultured bacterium clone SJ19 dinitrogenase reductase (*nifH*) gene, partial cds (97%)
16	2008 and 2009	AY630757.1	Uncultured bacterium clone SJY-2 dinitrogenase reductase gene, partial cds (100%)
17	2008 and 2009	GU083832.1	*Rhizobium giardinii* strain ZW7-1 nitrogenase reductase (*nifH*) gene, partial cds (99%)
18	2008 and 2009	EU770974.1	*Mesorhizobium septentrionale* CCBAU:03133 nitrogenase iron protein (*nifH*) gene, partial cds (100%)
19	2009	DQ995931.1	Uncultured nitrogen-fixing bacterium clone 67 dinitrogenase reductase (*nifH*) gene, partial cds (97%)
20	2009	AY907474.1	*Rhizobium gallicum* bv. *gallicum* strain IE988 nitrogenase reductase (*nifH*) gene, partial cds (98%)
21	2009	AY601060.1	Uncultured bacterium clone Langqian-3 dinitrogenase reductase (*nifH*) gene, partial cds (97%)
22	2009	DQ995922.1	Uncultured N_2_-fixing bacterium clone 57 dinitrogenase reductase (*nifH*) gene, partial cds (98%)
23	2009	GQ503352.1	*M ciceri* strain Rcd301 dinitrogenase reductase (*nifH*) gene, partial sequence (100%)

* Sequence similarity values below 97% are not considered to be identical (Stackebrandt and Goebel 1994).

† Sequences belonging to the strains of the commercial inoculant used.

### Distribution of *nifH* gene in chickpea rhizosphere as affected by treatments

Results of CA indicate that both disease control treatments and cultivars influenced the distribution of dominant *nifH* genes (Fig. 1). A significant relationship was found between *nifH* gene sequences and the combinations of disease control and cultivar treatments in 2008 (*P*=0·014). The sequence related to Clone b1-HA3-7 (sequence designation no. 5 as shown in Table 2) was associated with CDC Luna treatment III and the sequence related to *Azospirillum* sp. (sequence designation no. 10), with CDC Luna treatment II. The *nifH* gene sequence closely affiliated to *Azorhizobium caulinodans* (sequence designation no. 11) was frequent in the rhizosphere of CDC Vanguard control, but rare in the rhizosphere of CDC Luna (Fig. 1a). In 2009 (Fig. 1b), the relationship between *nifH* gene distribution and treatments was nonsignificant.

The *nifH* gene related to *M ciceri* contained in the commercial inoculant applied was frequently detected in the rhizosphere of protected CDC Vanguard in 2008, but rarely detected in CDC Luna rhizosphere (Fig. 1). The *nifH* gene with high similarity to *Bradyrhizobium* sp. contained in the inoculant was frequent in the rhizosphere of CDC Luna, but rare in that of CDC Vanguard, in both 2008 and 2009.

### Chemical disease control effects on biological N_2_ fixation

anova results showed that chickpea nodulation scores were significantly decreased with an increase in fungicide application intensity (Table 3), indicating that disease control treatments reduce chickpea nodulation. The concurrent enhancing effect of disease control on BNF (Table 3) suggested that disease control negatively impacted nodule size but not their functions. Nodule scores and N_2_-fixation were higher in 2008 than in the drier 2009 (Table 3).

**Table 3:** Effects of cultivar, disease control strategy, year and their interacting effects on nodulation scores, fixed N and grain yield in chickpea field, according to anova

Factors	Nodulation scores		Fixed N (kg ha^−1^)		Yield (kg ha^−1^)	
			
	Mean ± SE	*P*-value	Mean ± SE	*P*-value	Mean ± SE	*P*-value
Cultivar (C)						
Luna	6·4 ± 0·3	ns*	12·5 ± 1·5	<0·001	1357 ± 93	<0·001
Vanguard	6·9 ± 0·2	18·6 ± 1·5	1908 ± 90			
Disease control (D)						
Control	7·6 ± 0·3	<0·001	12·4 ± 1·9	0·04	1339 ± 124	<0·001
I	6·5 ± 0·3	17·2 ± 2·1	1630 ± 124			
II	5·8 ± 0·4	17·1 ± 2·3	1668 ± 133			
III	/	15·6 ± 2·6	1892 ± 176			
Year (Y)						
2008	7·0 ± 0·2	0·047	21·4 ± 1·4	<0·001	2030 ± 91	<0·001
2009	6·3 ± 0·3	9·7 ± 0·9	1235 ± 57			
C × D	/	ns	/	ns	/	ns
C × Y	/	ns	/	ns	/	ns
D × Y	/	ns	/	ns	/	<0·001
C × D × Y	/	ns	/	ns	/	0·02

* ns means nonsignificant at α = 0·05; *N *=* *64.

### Chemical disease control effects on disease expression and yield of chickpea

**Figure 2:**
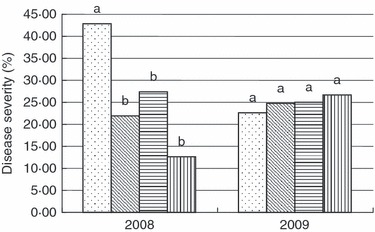
Effects of disease control application on disease severity in chickpea field in 2008 and 2009. (*P*=0·003 and 0·719 in 2008 and 2009). Different low case letters indicates significantly different means, according to Wilks’ Lambda test (*α*= 0·05, *n*=16). (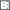) Control; (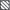) Treatment I; (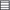) Treatment II and (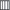) Treatment III.

A significant negative correlation between yield and disease severity in 2008 (Table 4) revealed the importance of disease outbreak as a yield limiting factor that year. Strong disease control × year interactions influenced yield (Table 3) reflecting that Ascochyta blight impacted plant productivity only in 2008 (Fig. 2), when wetter weather was conducive to early disease outbreak. In 2009, low disease pressure made disease control useless and no effect of chemical disease control strategies on disease severity was detected (Fig. 2). By contrast, no disease control × year interaction was found to influence nodulation score (Table 3), suggesting that fungicide application per se, rather than disease control, is the cause of reduced nodulation scores in fungicide-treated plants.

**Table 4:** Relationships among grain yield, fixed N and disease rate in chickpea field in 2008 and 2009, according to linear regression analysis

	2008			2009		
		
	Yield	Fixed N	Disease	Yield	Fixed N	Disease
Yield	1·000			1·000		
Fixed N	0·515**a	1·000		0·897**	1·000	
Disease	−0·761**	−0·263^ns^	1·000	−0·161^ns^	−0·145^ns^	1·000

a** Means *P *<* *0·001; *N *=* *32.

## Discussion

This study revealed an important diversity of *nifH* gene related to free-living diazotrophs in chickpea rhizosphere. Most of *nifH* gene sequences affiliated to N_2_-fixing bacterial species detected in the chickpea rhizosphere were uncultured and nonsymbiotic, indicating that free-living N_2_-fixing bacteria may also be involved in N cycling in Canadian Prairie agroecosystems. *Azohydromonas australica* was reported earlier as free-living N_2_-fixing bacteria in sorghum field (Xie and Yokota 2005) and isolated later as endophytic bacteria from storage root of sweet potato (Terakado-Tonooka *et al.* 2008), and *Sphingomonas azotifigens* was reported as free-living N_2_-fixing bacteria in rice fields (Xie and Yokota 2006). The presence and contribution of these free-living N_2_-fixing bacteria to BNF in chickpea field is not documented. The free-living bacteria *Azospirillum* sp., however, was recently reported in chickpea field, where they fixed N_2_ and promoted plant growth when co-inoculated with *Azotobacter* spp. and *Pseudomonas* spp. (Rokhzadi and Toashih 2011).

The nifH gene diversity in the fields studied may be larger than reported here.

Sequence analysis of the bands excided from the DGGE gel gave an insight into the dominant microbial taxa. Results presented should represent only the tip of the ‘iceberg’ of *nifH* gene diversity in chickpea rhizosphere, which would be revealed by more sensitive methods of massively parallel sequencing.

Our results suggests a contribution of the free-living N_2_-fixing bacterial community to the growth of chickpea mediated through BNF, but also through the promotion of other plant growth promoting bacteria. Plants strongly compete with micro-organisms for nitrogen (Hodge *et al.* 2000) and an actively growing crop plant may importantly reduce soil N availability to micro-organisms. However, free-living N_2_-fixing bacteria may reduce N starvation in the rhizosphere microbial community of actively growing crop plants.

The effects of disease control on BNF can be direct or indirect. Headline® Duo is a systemic fungicide, that is, it is absorbed by leaves and systemically moves within the plant. In our study, rhizosphere soil samples contained sizeable residual amounts of boscalid, an active ingredient of Headline® Duo. Disruption of the electron respiration chain in microbial cells by boscalid (Wang *et al.* 2009b) and pyraclostrobin (Bartlett *et al.* 2002), the other active ingredient of Headline® Duo, has been reported.

Whereas, fungicide application impacts the rhizosphere N_2_-fixing community (Gaind *et al.* 2007), here, this community appeared to be only mildly influenced. Based on MRPP analysis, application of Bravo® and Headline® Duo had insignificant influence on the diversity of *nifH* gene. However, CA detected changes induced by disease control on the structure of the *nifH* gene diversity in 2008, when control plants were severely impacted by Ascochyta blight, but not in 2009, which was dry in early summer and when the disease appeared only late in the season. The production of bioactive volatile compounds by chickpea leaves and roots was much higher in diseased than in fungicide protected chickpea in 2008 (A.F. Cruz, unpublished data), supporting the involvement of plant defence mechanisms rather than a direct effect of fungicide on *nifH* gene diversity. Thus, it seems that disease control is responsible for the differences found in the structure of the N_2_-fixing communities between protected and control rhizospheres in chickpea. Studies have shown that the composition of N_2_-fixing bacterial communities is affected by both soil conditions (Fierer and Jackson 2006) and their associated plant (Normand *et al.* 2007; Wang *et al.* 2009a).

Nodulation, in contrast to the rhizosphere N_2_-fixing bacterial community, seemed directly impacted by the fungicide application. Disease control reduced nodule size similarly in both years whether or not Ascochyta blight influenced the host plant. This concurs with former research showing fungicide-related modification in the rhizosphere in response to changes in plant photosynthesis (Petit *et al.* 2008), morphology (Baby *et al.* 2004) and root growth reduction (Ferreira *et al.* 2008). Fungicide-treated plants fixed more N_2_ than nontreated plants in our study; thus, the presence of small nodules, here, does not reflect reduced N_2_-fixing activity in protected plants. It may indicate that plants had allowed more bacteria entry in the recent past, perhaps after an episode of nodule shedding upon fungicide application, or that chemical protection influenced the process of nodulation in a way that increased the number of points of entry of symbiotic bacteria. Increased or changed chemical composition of protected plants root secretions could explain the changes observed in the composition of the rhizosphere N_2_-fixing bacterial communities of chickpea. Legumes produce specific chemical signals influencing symbiotic N_2_-fixing bacteria (Geurts *et al.* 2005) and perhaps other bacteria.

Genotype effects on N_2_-fixing bacterial community in chickpea rhizosphere were important and confirm the results of a previous study on host range in rhizobium isolates (Ampomah *et al.* 2008). The selective effects of genotype on N_2_-fixing bacteria could be owing to the differences in root secretion between cultivars, as proposed earlier (Lupwayi and Kennedy 2007). The growth and population densities of rhizosphere bacteria can be increased by large amounts of root secretion, sloughing-off of root cap cells and senescing root epidermis in rhizosphere soil (Nguyen 2003). The symbiotic N_2_-fixing bacteria could also be influenced by differences in the symbiotic signalling physiology of the two chickpea genotypes. Specific flavonoids produced by legumes attract specific rhizobia to their root hairs, and the rhizobia in turn, produce the ‘nod factors’ that induce root hair infection and nodule formation (Geurts *et al.* 2005). Differences in the signalling system of different chickpea genotypes could result in differences in the nodulation pattern between the plants or in the structure of the N_2_-fixing bacterial communities in their rhizosphere.

Foliar fungicide application to control Ascochyta blight in chickpea crop is a widespread agronomic practice. Overall, the results of DGGE and clone libraries revealed that disease control strategies can modify nodulation and the composition of the nitrogen fixation associated gene fragments in rhizosphere apparently through its effect on the crop plants. This effect of disease control strategies tested in our study was relatively small and may have been modified by environmental conditions. Environmental influences have lower impact than fungicide application on the process of nodulation, which is more intimately related to the plant than rhizosphere composition and differently regulated. Environmental conditions, conducting to disease, trigger plant defence reactions seemingly impacting free-living N_2_-fixing bacteria.
